# Developing a wintering waterfowl community baseline for environmental monitoring of Narragansett Bay, Rhode Island

**DOI:** 10.12688/f1000research.6080.2

**Published:** 2015-09-24

**Authors:** Betty J. Kreakie, Kristopher Winiarski, Richard McKinney

**Affiliations:** 1 U.S. Environmental Protection Agency, Office of Research and Development, Atlantic Ecology Division, Narragansett, RI, 02882, USA; 2 Department of Environmental Conservation, 160 Holdsworth Way, University of Massachusetts, Amherst, MA, 01003, USA

**Keywords:** Baseline Data; Community; Environmental Monitoring; Narragansett Bay; Nonmetric Multidimensional Scaling; Waterfowl

## Abstract

In 2004, the Atlantic Ecology Division of the US Environmental Protection Agency’s Office of Research and Development began an annual winter waterfowl survey of Rhode Island’s Narragansett Bay. Herein, we explore the survey data gathered from 2004 to 2011 in order to establish a benchmark understanding of our waterfowl communities and to establish a statistical framework for future environmental monitoring. The abundance and diversity of wintering waterfowl were relatively stable during the initial years of this survey, except in 2010 when there was a large spike in abundance and a reciprocal fall in diversity. There was no significant change in ranked abundance of most waterfowl species, with only Bufflehead ( *Bucephala albeola*) and Hooded Merganser ( *Lophodytes cucllatus*) showing a slight yet significant upward trend during the course of our survey period. Nonmetric multidimensional scaling (NMDS) was used to examine the community structure of wintering waterfowl. The results of the NMDS indicate that there is a spatial structure to the waterfowl communities of Narragansett Bay and this structure has remained relatively stable since the survey began. Our NMDS analysis helps to solidify what is known anecdotally about the bay’s waterfowl ecology, and provides a formalized benchmark for long-term monitoring of Narragansett Bay’s waterfowl communities. Birds, including waterfowl, are preferred bioindicators and we propose using our multivariate approach to monitor the future health of the bay. While this research focuses on a specific area of New England, these methods can be easily applied to novel areas of concern and provide a straightforward nonparametric approach to community-level monitoring. The methods provide a statistic test to examine potential drivers of community turnover and well-suited visualization tools.

## Introduction

As modern environmental pressures continue to adversely impact natural habitats, global waterfowl populations are declining at accelerated rates. According to the Millennium Ecosystem Assessment, approximately 12% of all bird species are presently threatened with extinction, and 41% of all waterfowl populations are declining in abundance ( Assessment, 2003; Assessment, 2005). Narragansett Bay’s waterfowl communities are not immune to the global drivers of waterfowl population decline and modifications of waterfowl communities; Narragansett Bay is exposed to habitat conversion, shoreline hardening, increased sedimentation and pollution, and increased threats from climate change ( Nixon & Fulweiler, 2012). Even though we know that these changing conditions are having a global impact on waterfowl populations, we are uncertain about the specific ramifications on Narragansett Bay’s wintering waterfowl communities. Twenty-three North American waterfowl species have been observed wintering in Narragansett Bay, including 11 of the 15 known species of sea ducks, a guild of waterfowl that breed in boreal Canada and winter as far south as Chesapeake Bay ( McKinney, 2004). Understanding or predicting deviations from normal is not possible without baseline monitoring data on waterfowl communities ( Temple & Wiens, 1989).

In 2004, the Atlantic Ecology Division (AED) of the US Environmental Protection Agency’s (EPA’s) Office of Research and Development, in collaboration with state wildlife agencies and local environmental groups, began an annual winter waterfowl survey of Rhode Island’s Narragansett Bay (hence forth referred to as the Bay). Every year in January, local wildlife biologists and environmental scientists conduct a comprehensive survey of the Bay’s waterfowl. The waterfowl survey was implemented in an attempt to fill critical gaps in our ecological knowledge about the Bay’s waterfowl communities. While there have been numerous waterfowl studies conducted in this area ( Caron & Paton, 2007; Loring *et al.*, 2013; McKinney & McWilliams, 2005), we are unaware of any long-term multispecies studies. Consequently, we are still relatively ill-informed about long-term trends of the Bay’s waterfowl populations and communities. The survey data can also provide us a means to monitor the Bay’s overall environmental health by using waterfowl as a bioindicator. Due to waterfowl’s comparatively high trophic status, waterfowl communities provide insight about local food webs’ relative health and stability.

To determine the underlying waterfowl community structure, we used a multivariate ordination technique, Nonmetric multidimensional scaling (NMDS) ( Austin, 1976; Clarke, 1993). Additionally, we propose employing the NMDS as a statistical framework for environmental monitoring of the Bay ( Gabrey & Afton, 2004; Urban, 2006). Like other ordination methods, the NMDS approach reduces data complexity, which is critical when analyzing data that are complex and highly variable. Yet unlike other ordination methods, NMDS requires few, if any, a priori assumptions about the distribution of the data. This multivariate approach allows us to detect any relative shifts in community composition between sites and years, and also to explore relationships with potential environmental drivers of change ( Clarke & Ainsworth, 1993; Karydis, 1992).

Birds, especially waterfowl, are often the logical candidates for monitoring environmental health ( Amat & Green, 2010; Kushlan, 1993). Since waterfowl are high trophic level foragers, the environmental stressors of all lower trophic levels accumulate in waterfowl ( Matsinos & Wolff, 2003; O’Connell *et al.*, 2000). Waterfowl have, therefore, been used successfully to monitor a wide array of environmental stressors. For example, these species have been used to monitor a range of heavy metals, including cadmium, mercury, and lead ( Di Giulio & Scanlon, 1984; Mochizuki *et al.*, 2002; Monteiro & Furness, 1995; Ribeiro *et al.*, 2005), and the impacts of habitat conversion, e.g., forested land to agriculture or road ( Austin, 1976; Koper & Schmiegelow, 2006; Maisonneuve *et al.*, 2006). Birds respond to habitat conversion at multiple temporal and spatial scales ( DeLuca *et al.*, 2004). Waterfowl respond to these stressors at the local up to the regional scale and their responses can be apparent nearly immediately and continue after substantial time lags ( Findlay & Bourdages, 2000). These species have a high detection probability and are easy to identify by even novice birders ( Pagano & Arnold, 2009), which further strengthen the argument of using waterfowl as bioindicators of environmental health.

It is nearly impossible to overstate the economic and environmental significance of Narragansett Bay to New England. Narragansett Bay contributes meaningfully to the economy through recreation, tourism, fishing, and shipping ( Pastore, 2011; Tyrrell *et al.*, 1994). Its economic contributions are equally matched by its environmental contributions. The Bay serves as critical habitat to numerous species and provides innumerable ecosystem services. This study analyzes the first eight years of our survey data in order to develop a baseline understanding of waterfowl community spatial and temporal structure in the Bay, which can be used to track future changes in the overall health of the Bay.

## Methods

### Study site

**Figure 1.**
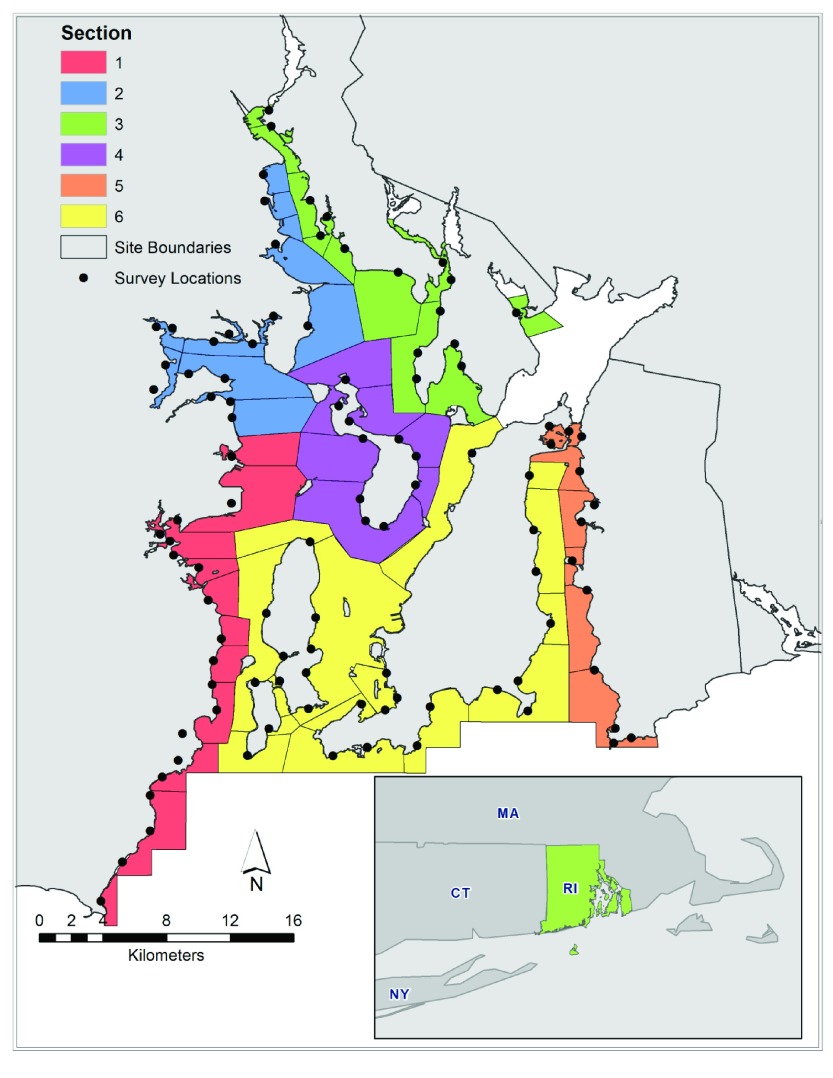
Map of Narragansett Bay, Rhode Island. Inset of Rhode Island (USA). Sections are color-coded and site boundaries delineated. The points represent the approximate observation locations for the surveys.

Narragansett Bay is a well-mixed embayment containing a complex of estuaries ( Figure 1). The Bay is 234 km ^2^ in area and has a mean depth of 8.7m ( Calabretta & Oviatt, 2008; Nixon *et al.*, 2009). In the northern portion of the Bay, the Seekonk and Providence Rivers are the main freshwater sources. This area is also more urban, and exposed to periodic hypoxia, especially in the summer months ( Codiga *et al.*, 2009). The southern portion of the Bay is deeper and has more intense oceanic influences. Additionally, the land surrounding the southern portion tends to be less densely developed and populated.

### Survey method

Initiated in 2004, the NBWWS is completed annually by eight teams composed of 2–4 observers who survey waterfowl at 67 site locations throughout Narragansett Bay ( McKinney, 2004). Survey locations were determined by dividing the bay first into sections, and then further into sites within the sections ( Figure 1). The number of sites in each section ranged from eight sites in sections 4 and 5 to 15 sites in section 6. The division of sections and sites was based on the geography of the Bay; this layout ensures that all areas of the Bay will be visible from the survey locations ( Figure 1).

Coordinated sampling occurs at all sites during a single day in early to mid-January, beginning at approximately 0730 in the morning and ending by 1645 in the afternoon. This is a shore-based survey and observers use direct counts to record all waterfowl present at a location at the time of the observation. We define waterfowl species as ducks, geese, swans, and grebes. All birds were identified to the species level, except Lesser and Greater Scaups, that were simple categorized into a single Scaup (Aythya spp.) taxonomic group due to the difficulty of distinguishing them.

Counting is completed from a stationary point from which the entire area (i.e., cove or embayment) is scanned with binoculars or a spotting scope. Every bird seen on the water surface or on the adjoining shoreline up to 50 m from the water is counted; when possible sex and age were also noted. Large flocks of greater than 100 birds are estimated by counting in groups of ten or one hundred. Observers take as much time as necessary to accurately count and record all waterfowl observed. Most locations require between 10–20 minutes to survey.

### Data analysis

We initially explored basic population trends for each waterfowl species. This included average abundance and standard deviations between years. We calculated ranked abundance for non-rare species and fitted regression trends and tested significance for each species. Ranked abundance allowed us to examine the relative dominance or rarity of a species given the current community. Furthermore, we assessed total waterfowl abundance and Shannon diversity index by year.

We used nonmetric multidimensional scaling to identify the community level structure of wintering waterfowl in Narragansett Bay ( Austin *et al.*, 2001; Clarke & Ainsworth, 1993; Kruskal, 1964). Nonmetric multidimensional scaling is a nonparametric ordination technique used to reduce the dimensionality of a complex data set while maintaining the relative relationship between species composition of sites ( Dixon, 2009). This specific ordination method does not require a prior constraining of axes or assumptions of normality. NMDS fits all ordination axes simultaneously not by sequentially finding orthogonal linear axes. Therefore, calculating variance explained by axis or linear goodness of fit measures are not applicable to this specific ordination method.

Nonmetric multidimensional scaling was conducted using survey data collected from 2004 to 2011. A Wisconsin double transform, standardized by species percent abundance and by maximum for each species, was conducted on the species data, and Bray-Curtis distance was used to calculate community distance (for in-depth discussion of methods see Faith *et al.*, 1987; Minchin, 1987). We iteratively fit NMDS solutions of increasing dimensionality to determine the solution with adequate levels of decreased stress. Stress is a measure of goodness of fit; it is a measure of the agreement between the distance in ordination space and observed waterfowl community distance ( Kruskal, 1964). Our goal was to minimize stress while avoiding superfluous ordination axes. To find the global stress minimization of an NMDS, random configurations of start locations were interjected into the fitting algorithm ( Kruskal & Wish, 1978). All analyses were conducted in R version 2.13.1 ( R Development Core Team, 2013) and NMDS was conducted with the Vegan package ( Oksanen *et al.*, 2007).

To explore relationships between environmental conditions and the waterfowl community structure, we tested for correlations between the NMDS axes and ancillary variables ( Table 1). Ancillary variable selection was hypothesis driven. Initially, we examined the area effect and spatial structure of the waterfowl community composition. Also, we explored the impacts of near shore habitat conversion. Furthermore, we hypothesized that winter intensity and extreme wind events might be driving inter-annual variation. All ancillary variables ( Table 1) were initially tested for significance to the final four-dimensional NMDS structure and then cross correlation between variables. Among highly correlated variables, only the most significant variables to the NMDS structure were included in the final analysis.

**Table 1.** Ancillary variables.

Abbreviation	Description
**Latitude**	Latitude of site centroid
**Longitude**	Longitude of site centroid
**mean_bath**	Average bathymetry of site
**Depth**	Deepest point in the site
**low_bath**	Shallowest point in the site
**std_bath**	Standard deviation of bathymetry in the site
**Area**	Total area of the site
**Perimeter**	Total length of the site perimeter
**degr_wet_area**	Total area of wetlands classified as degraded within the site
**Wetland**	Total wetland area within the site
**NAO.1**	North Atlantic Oscillation Index for January of survey year
**NAO.12**	North Atlantic Oscillation Index for December prior to survey
**NAO.11**	North Atlantic Oscillation Index for November prior to survey
**NAO.win**	Average North Atlantic Oscillation Index for January, December, and November prior to survey
**NAO.10**	North Atlantic Oscillation Index for October prior to survey
**NAO.9**	North Atlantic Oscillation Index for September prior to survey
**NAO.8**	North Atlantic Oscillation Index for August prior to survey
**NAO.fall**	Average North Atlantic Oscillation Index for October, September, and August prior to survey
**WS_day**	Wind speed the day of survey
**WS_day_b4**	Wind speed the day before survey
**WS_3day**	Average wind speed for the three days prior to survey
**WS_7day**	Average wind speed for the seven days prior to survey
**WS_30day**	Average wind speed for the thirty days prior to survey
**WD_day**	Wind direction the day of survey
**WD_day_b4**	Wind direction the day before survey
**WD_3day**	Average wind direction for the three days prior to survey
**WD_7day**	Average wind direction for the seven days prior to survey

Location was measured as the latitude and longitude of each site’s centroid. Delineated site boundaries were used to calculate area and site perimeter length ( Figure 1). Degraded wetland area was calculated using RI Department of Environmental Management (RI DEM) Statewide Planning Program’s impacted wetland digital vector data, which were downloaded from the RI Geographic Information System (RIGIS) ( http://www.edc.uri.edu/rigis). Total wetland area was calculated from the US Fish and Wildlife Service (US FWS) National Wetland Inventory ( http://www.fws.gov/wetlands). National Oceanic and Atmospheric Administration’s (NOAA) bathymetry data were also downloaded from RIGIS.

The North Atlantic Oscillation (NAO) is a large-scale climate index that measures the atmospheric pressure at sea level between the Icelandic low and Azores high, which captures information about the relative intensity of the winter ( Hurrell, 1995). A strongly positive NAO index is related to above normal temperatures in the study region, whereas a negative NAO index is associated with colder, more severe winters ( Visbeck *et al.*, 2001). The NAO index data were obtained from the National Center for Atmospheric Research (NCAR) ( climatedataguide.ucar.edu). We used the NAO index to investigate whether winter waterfowl habitat selection was impacted by the relative severity of the winter weather. Wind speed variables were calculated from data downloaded from the NOAA National Climatic Data Center ( http://www.ncdc.noaa.gov).

## Results

Environmental data Ancillary variables: The abbreviation definitions can be found in Table 1. Click here for additional data file. Copyright: © 2015 Kreakie BJ et al. 2015 Data associated with the article are available under the terms of the Creative Commons Zero "No rights reserved" data waiver (CC0 1.0 Public domain dedication).

Winter waterfowl survey Species data: The abbreviation definitions can be found in Table 2. Click here for additional data file. Copyright: © 2015 Kreakie BJ et al. 2015 Data associated with the article are available under the terms of the Creative Commons Zero "No rights reserved" data waiver (CC0 1.0 Public domain dedication).

**Figure 2.**
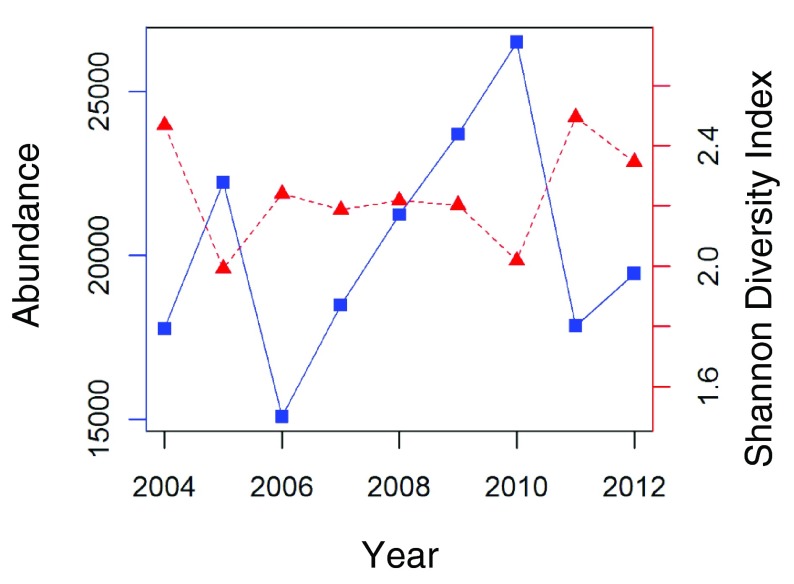
Waterfowl abundance and Shannon diversity index.

**Figure 3.**
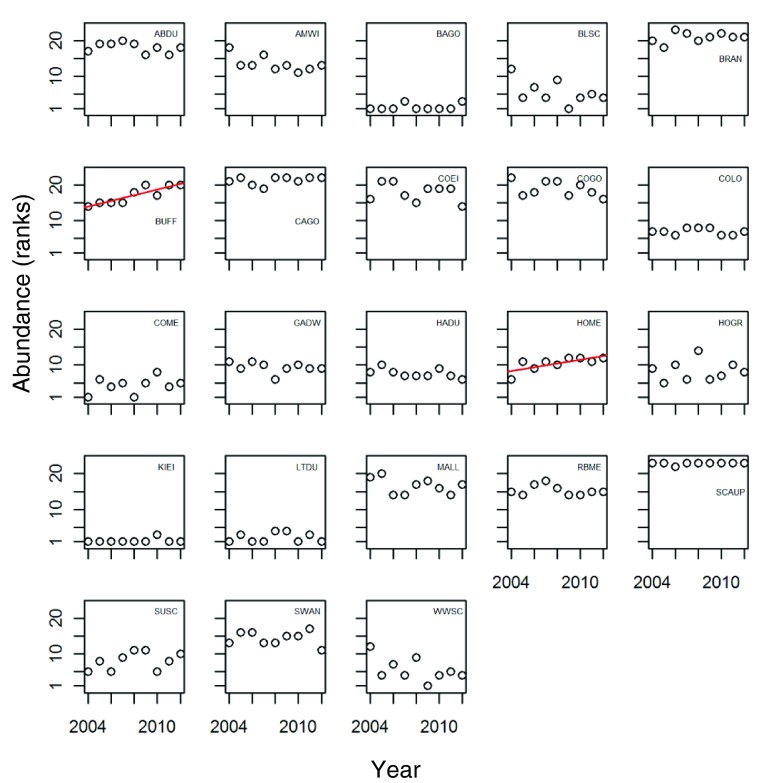
Non-rare species ranked abundances.

A total of 23 waterfowl taxa were recorded and included in the analysis presented within this study ( Table 2). The total waterfowl population for the Bay averaged approximately 20,000 individuals annually. Total count was lowest in 2006 (15,090 individuals) and highest in 2010 (26,503 individuals) ( Figure 2). The 2010 peak in abundance was due to a spike in the number of Scaup spp. present in the Bay. This jump in Scaup spp. abundance corresponded to a reciprocal dip in the 2010 Shannon diversity index ( Figure 2). Ranked abundances of individual species showed no significant trends of increase or decrease over the course of this study, except for slight increases in Bufflehead ( *Bucephala albeola*) and Hooded Merganser ( *Lophodytes cucullatus*) ( Figure 3).

**Table 2.** Species summary of the Narragansett Bay Winter Waterfowl Survey for 2004–2011. Mean is the average abundance for each species throughout the entire study area. Percent represents the fraction that each species contributes to the total for duration of the study. *Species making up less than 1% of the community, and considered rare. Trend analysis ( Figure 3) were not conducted on these rare species.

Species	Species Code	Mean (+/-) SD	Percent
**American Black Duck** *Anas rubripes*	ABDU	1205 ± 176	5.95
**American Wigeon** *Anas americana*	AMWI	456 ± 334	2.25
Barrow’s Goldeneye* *Bucephala islandica*	BAGO	0.3 ± 0.7	~0
**Black Scoter** *Melanitta americana*	BLSC	316 ± 355	1.56
**Brant** *Branta bernicla*	BRAN	2525 ± 881	12.5
**Bufflehead** *Bucephala albeola*	BUFF	1052 ± 431	5.19
**Canada Goose** *Branta canadensis*	CAGO	2713 ± 1249	13.4
**Common Eider** *Somateria mollissima*	COEI	1302 ± 579	6.43
**Common Goldeneye** *Bucephala clangula*	COGO	1374 ± 532	6.78
Common Loon* *Gavia immer*	COLO	67 ± 41	0.332
Common Merganser* *Mergus merganser*	COME	26 ± 23	0.128
Gadwall* *Anas strepera*	GADW	144 ± 105	0.712
Harlequin Duck* *Histrionicus histrionicus*	HADU	71 ± 22	0.351
Hooded Merganser* *Lophodytes cucullatus*	HOME	171 ± 110	0.842
Horned Grebe* *Podiceps auritus*	HOGR	138 ± 185	0.682
King Eider* *Somateria spectabilis*	KIEI	0.1 ± 0.3	~0
Long-tailed Duck* *Clangula hyemalis*	LTDU	1 ± 2	~0
**Mallard** *Anas platyrhynchos*	MALL	1002 ± 410	4.94
**Red-breasted Merganser** *Mergus serrator*	RBME	771 ± 205	3.8
**Scaup spp.** *Aythya spp.*	SCAUP	6146 ± 2750	30.3
Surf Scoter* *Melanitta perspicillata*	SUSC	81 ± 66	0.401
**Swan** *Cygnus spp.*	SWAN	618 ± 267	3.05
White-winged Scoter* *Melanitta fusca*	WWSC	78 ± 130	0.386

**Figure 4.**
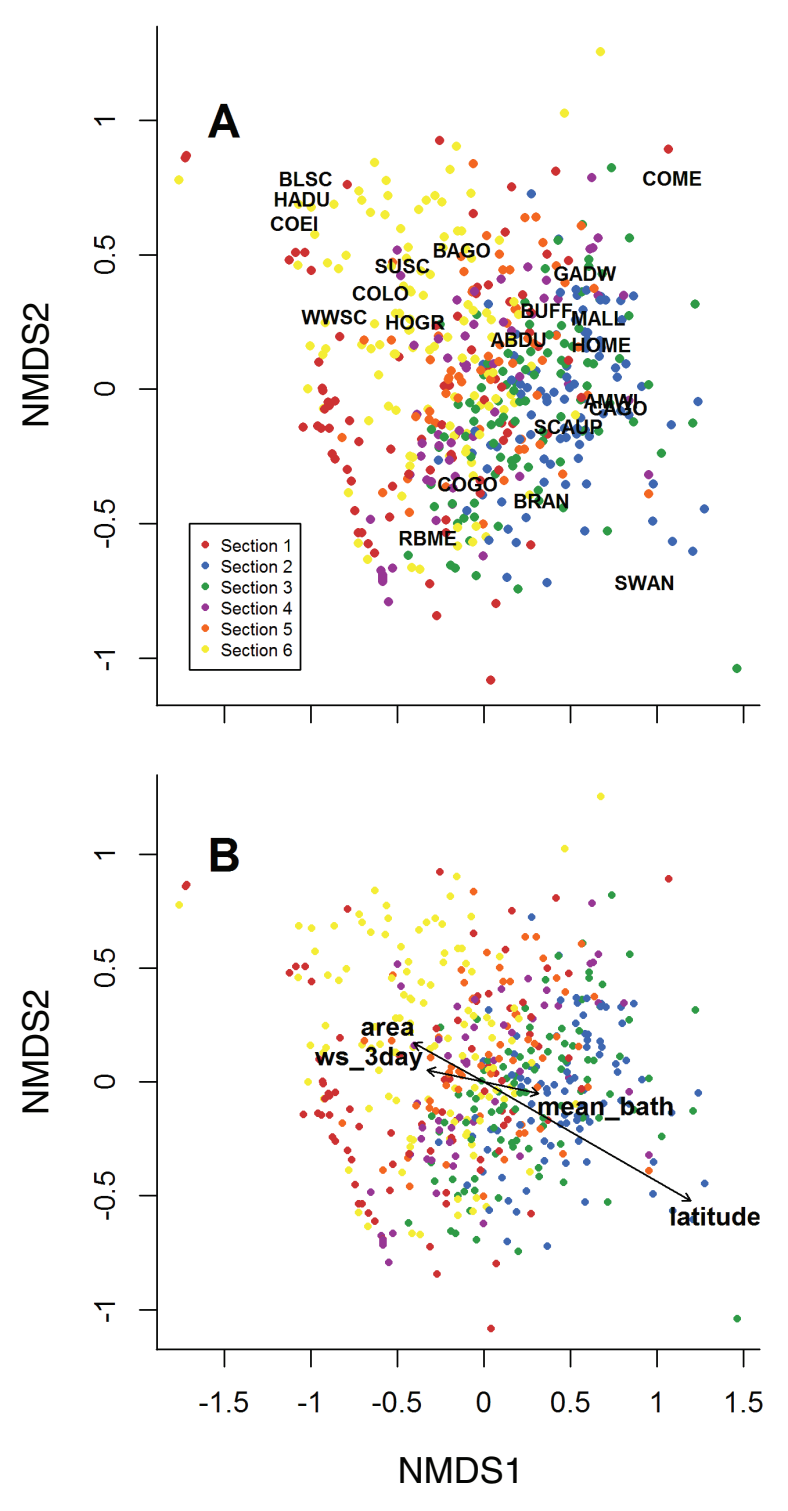
Final NMDS results. The circles illustrate the location of a single site for each surveyed year. The sites are color-coded by section. Figure 4A: Species locations are illustrated with four-letter abbreviation. Figure 4B: Bi-plot of NMDS axis one and two with vectors of significant environmental variables overlaid. The arrow’s direction illustrates the environmental gradient and the length is proportional to the correlation strength between the variable and the NMDS (See Table 3).

Our final NMDS fit had four dimensions and a stress value of 0.1449 ( Figure 4A), and is well within the acceptable stress limits of NMDS ( Clarke, 1993; Kruskal & Wish, 1978). Sites in the NMDS plot that are closer in ordination space are more similar in species composition. As the distance between points increases, the species composition becomes more dissimilar. Our final presentation of the NMDS rotated the data so that greatest distance between site scores are plotted on NMDS axis 1. There is some spatial clustering of survey sections across the first two NMDS axes. Sections 1 and 6 are concentrated on the left side of the NMDS axis 1, while sections 2 and 3 are predominantly on the right side. Sections 4 and 5 are located approximately in the middle, as they are in actual Bay position.

**Table 3.** Results of final NMDS.

Variable	NMDS 1	NMDS 2
Latitude***	0.916	-0.401
mean_bath**	0.987	-0.160
Area***	-0.920	0.392
**NAO.12**	0.247	-0.969
**NAO.11**	-0.0618	0.998
**NAO.win**	0.145	-0.989
WS_3day**	-0.988	0.157
**WS_30day**	0.600	0.799
WD_day**	0.563	-0.826
WD_day_b4*	-0.335	0.942

Significance codes: “***” < 0.001, “**” <0.01, “*” <0.05

Species location in the ordination space can approximate how species sort into habitat types along the spatial gradient in the Bay ( Figure 4a). The upper left of the ordination space reflects predominantly open ocean, deep-water sites (sections 1 and 6). In line with the habitat location in ordination space, indicator species of deeper water oceanic habitat (e.g. Harlequin Duck ( *Histrionicus histrionicus*) and Common Eider ( *Somateria mollissima*)) are also positioned in the upper left hand corner of the ordination. Northern sites in the Bay are more shallow and marsh-like (sections 2 and 3), with species indicative of this habitat type (e.g. Atlantic Brant ( *Branta bernicla*) and Canada Goose ( *Branta canadensis*)).

After the removal of correlated variables, ten ancillary variables were fit to the NMDS results ( Table 3). Only latitude, mean bathymetry, area, and the average wind speed three days prior to the survey were significantly correlated to the NMDS ( Figure 4B). Wind speed the day of the survey and day before were slightly less significant. Although not significant, the NAO index for November, December, and winter average showed strong relationships with NMDS axis 2.

**Figure 5.**
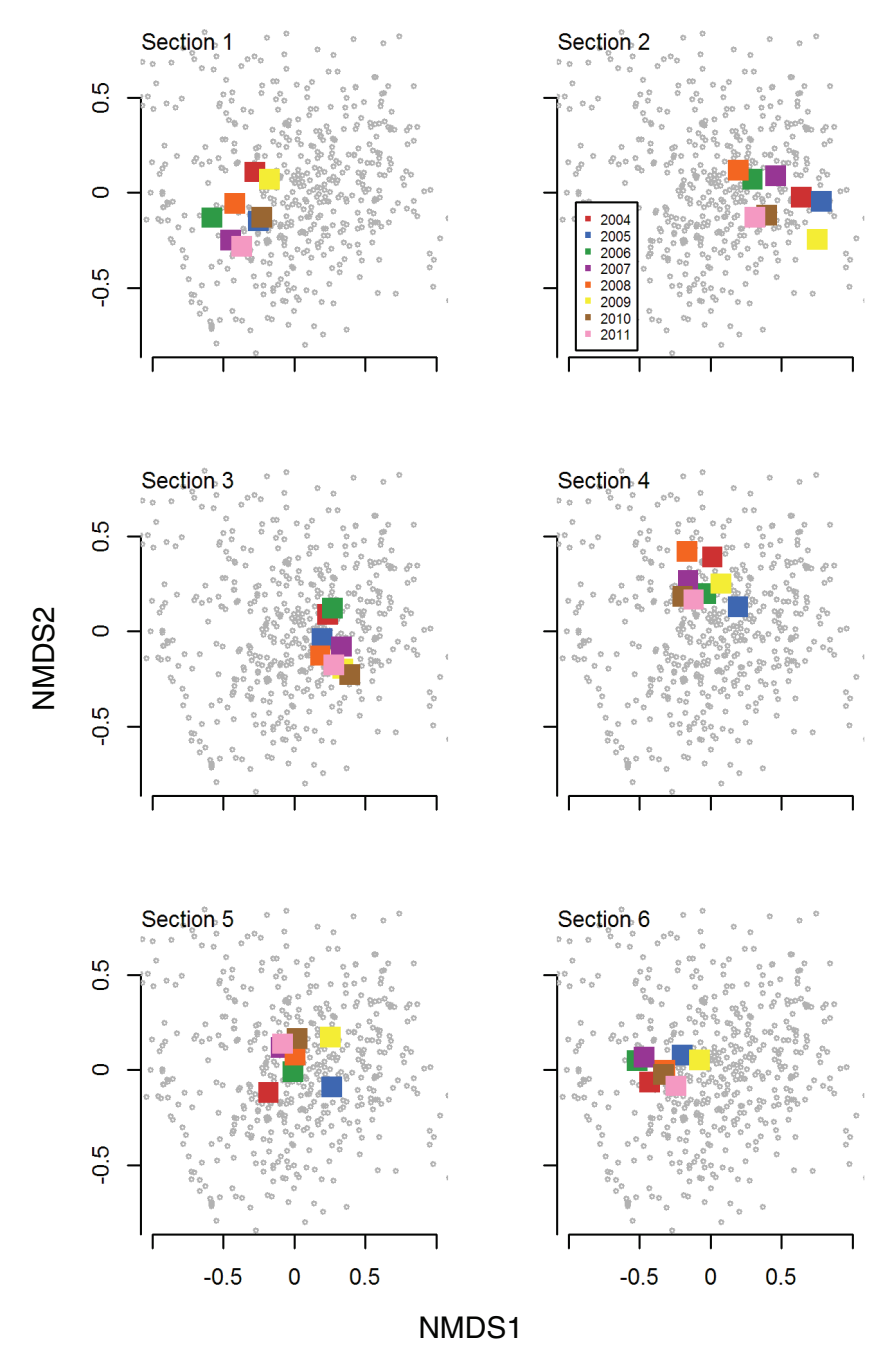
Final NMDS illustrating average community ordination location of section by year. Illustrates where the section exists in the overall ordination space and how that position changes through the study period.

Most sections’ locations in the ordination space did not change drastically between years; they remained in the same relative location of the ordination ( Figure 5). The relative stability of sections in ordination space among years indicates that communities had consistent species composition between years. Section 2 shifted the most among years, especially between 2008 and 2009 ( Figure 5). Additionally, when compared to the other sections, section 2 had the greatest between group average distances through the duration of this study.

## Discussion

Our community level analysis allowed us to establish the expected spatial and temporal structure of the Bay’s winter waterfowl communities which can be used to monitor future environmental changes. Spatially, the NMDS analysis formalized several aspects of the Bay’s waterfowl community that were previously understood only anecdotally, and provided a clear depiction of the community spatial structure across the Bay. NMDS as a monitoring tool in conjunction with the baseline conditions identified in this study could be particularly useful in identifying any future changes in waterfowl community structure in this region and change in the Bay’s overall health.

In the upper portions of the Bay (sections 2 and 3), waterfowl habitat is characterized by shallow, salt-marsh dominated coves and sheltered coves and shorelines with ample freshwater inputs. These sites mapped predominantly on the right-side of the NMDS ( Figure 4). Dabbling duck species such as Mallard ( *Anas platyrhynchos*) and American Black Duck ( *Anas rubripes*) use salt-marsh sites at high tide for cover, protection from predators, and feeding, and as sites for roosting at low tide ( Bellrose, 1980). Several smaller diving ducks, including benthic-feeding Bufflehead and piscivorous Hooded Merganser use sheltered coves and shorelines for feeding during the day ( McKinney, 2004). Canada Goose and Mute Swan ( *Cygnus olor*) also utilize these sites as they presumably provide ready access to submerged aquatic vegetation on which these species feed ( Mowbray *et al.*, 2002). An urban center (city of Providence) and several expansive suburban cities are located in close proximity to the upper Bay sites, and it is in this region of the Bay where urban development would be expected to most influence waterfowl distribution.

The middle portion of the Bay is characterized by an increase in deeper, open water habitats, which continue towards the Bay mouth where they are supplemented by rocky shoreline habitats. These sites concentrated on the left-side of the NMDS ( Figure 4, color-coded red and yellow). Open-water species such as Red-breasted Merganser ( *Mergus serrator*), Common Goldeneye ( *Bucephal clangula*), Scoter species ( *Melanitta* spp.), and Common Eider use this region of the Bay. These species have larger body sizes that enable them to dive in deeper water to take advantage of prey not found in shallow water areas (e.g., Blue Mussel, *Mytilus edulis*), and of benthic prey such as crabs that migrate to deeper water during winter ( Ehrlich *et al.*, 1988). Harlequin Duck uses rocky shoreline habitats found near the mouth of the Bay that reflect their northern rocky stream breeding sites and where they can feed on benthic invertebrates such as amphipods ( Robertson & Ian Goudie, 1999). The lower Bay sites are currently less impacted by adjacent urbanization effects as shorelines tend to be more sparsely populated. However, knowledge of baseline conditions could lend insight into interpreting data from future monitoring and aid in identifying any impacts of increased urbanization, or changes in waterfowl community structure resulting from displacement of upper Bay species.

In addition to species’ life history strategies, climatic factors can potentially influence waterfowl distribution in estuaries. In our study, wind speed, averaged over the three day period before sampling, was the only significant dynamic variable included in the final NMDS. This too reinforces what was communicated anecdotally about the movement of waterfowl through the Bay. Large groups of birds will shift their location in the Bay during prolonged high wind events, such as nor’easters. Typically before and during large storms, birds will relocate to the leeward side of islands or into wind protected coves. Due to global climate change, nor’easter frequency and intensity have increased on a global scale ( Yohe & Hope, 2013). It is assumed that Narragansett Bay will be impacted by this increase in predicted probability of severe winter events. Our study suggests waterfowl respond to short-term changes in wind speed, and it will be of interest to monitor how waterfowl respond to any increase in both intensity and frequency of extreme wind events.

We observed a small, yet significant, increase in the ranked abundance of Hooded Merganser, and an even more apparent upward trend of the Bufflehead population. The Bufflehead trend may be the effect of increased level of protection for this species. In the early 20 ^th^ century, Bufflehead populations were in decline due to over harvest and in response received increased protection through such means as reduced bag limits ( Gauthier, 1993). In addition, Bufflehead nesting boxes were installed to compensate for loss of breading habitat ( Erskine, 1960; Owen & Black, 1990). Population numbers have been growing steadily since, and perhaps this rebound could explain the increasing trend we saw in Narragansett Bay. Since we used ranked abundance, we hypothesize that the elevated conservation status of Bufflehead and subsequent population growth is providing the Bufflehead a competitive advantage in the Bay. However, more research is needed to fully understand the dynamics and drivers of the changes in Bufflehead populations in the Bay.

As we move forward with our monitoring, the NMDS approach can provide a useful means to compare future survey data with baseline conditions established during the first ten years of the survey. The NMDS provides a statistical framework to analyze monitoring data at the site and section level, but in context of the entire Bay ( Faith *et al.*, 1987). Due to random noise, we expect the position of the site or the average position of the section (as in Figure 5) to undergo relatively small changes in ordination space from year to year. In contrast, locations that jump from one year to the next, or that display a trajectory through NMDS space through time may indicate environmental forcing that is more than random.

For example, the relatively large shifts of section 2 between years may be a characteristic of this particular section; it is thought that waterfowl communities in this part of the Bay vary greatly according to the intensity of winter and amount of ice. However, large shifts among years or over the course of several years may be an indicator of environmental change. Additionally, if we see more variability in the amount of ice in this section, it may be more difficult to define a collective waterfowl community for this section. The turnover between years might simply overwhelm any potential community signal. To highlight the effectiveness of the method for monitoring, we already know from this baseline analysis that section 2 is an area that requires special consideration in the future.

Although the NMDS shows promise as an effective statistical framework, there are obvious limitations to our approach. First and foremost, annual surveys provide discrete snapshots of the Bay’s waterfowl community through time. We survey one day a year, and only in the winter when these species are present. We do not have a quantitative estimate of the amount of variability data arising from either short-term (i.e., daily) or longer-term (weekly or monthly) movements of individuals among sites, or into and out of our study area. Because of this, our ability to detect change in community composition may require data collection over an extended time period. Yet despite these shortcomings, the ease of data collection and robustness of the NMDS method make it a viable long-term monitoring option.

In this study we proposed an approach to analyzing long-term waterfowl monitoring data in order to establish baseline conditions against which future trends in community composition and habitat utilization can be compared. This approach provides a quantitative yet visual means to represent baseline community structure and observed patterns of waterfowl distribution, and provides an easily interpreted series of templates against which future observations or patterns of change can be evaluated. Our approach was developed for waterfowl Narragansett Bay, but can be applied to other estuaries and potentially other species, although environmental factors in the model may need to be modified to reflect those relevant to the species investigated. Overall, our approach will help facilitate the use of waterfowl populations, as well as other relevant species, to monitor the environmental health of a large bay.

## Data availability

The data referenced by this article are under copyright with the following copyright statement: Copyright: © 2015 Kreakie BJ et al.

Data associated with the article are available under the terms of the Creative Commons Zero "No rights reserved" data waiver (CC0 1.0 Public domain dedication). 

F1000Research: Dataset 1. Environmental data, 10.5256/f1000research.6080.d42953 ( Kreakie *et al.*, 2014a).

F1000Research: Dataset 2. Winter waterfowl survey, 10.5256/f1000research.6080.d42954 ( Kreakie *et al.*, 2014b).
