# Supplementary material for: Developing a wintering waterfowl community baseline for environmental monitoring of Narragansett Bay, Rhode Island
Source: F1000Res. 2015 Sep 24;4:40. Originally published 2015 Feb 10. [Version 2] doi: 10.12688/f1000research.6080.2 (PMC4806708; doi:10.12688/f1000research.6080.2)
Supplement: Peer review history

# Peer review: Referee response for version 1

**DOI:** 10.5256/f1000research.6509.r7882

**Published:** 2015-07-27

**Authors:** José Manuel Arcos

**Affiliations:** 1 SEO/BirdLife, Barcelona, Spain

This is an interesting paper describing a waterfowl winter community and its evolution through time. Authors use non-metric dimensional scaling to assess changes in community structure, taking into account environmental features (habitat, climatic, metheorological). The work seems adequately conducted and is based on a sound dataset that extends along 8 years. I find therefore the work worthy to be indexed. However, I have some concerns about how the work is presented. Overall, it has a too local focus and should be put in a wider context, with clear aims. I hope the following comments, by sections, will help authors to improve the current version.

**Abstract**

This section is particularly too local it should give a wider view of the work and its potential contribution beyond the study area. The authors propose a methodological approach that could be extended to other areas, to solve similar questions (i.e. how to assess changes in wintering waterfowl communities over time, in a scenario of climate change and increasing coastal development). The last paragraph of the discussion is written under this philosophy, and part of it could be used here. Of course it’s also important to depict the results, but they need to be better contextualized.

Another issue to address is the excess of repetitions (the same problem is applicable to other sections of the manuscript). For instance, Narragansett Bay is quoted eight times in the abstract alone, and the analytical tool "nonmetric multidimensional scaling" is written in full three times (worth introducing here the acronym NMDS).

**Introduction**

Again I find the text too local. Only the first two sentences of paragraph 1, plus paragraph 4 attempt to put the study in a wider context. I suggest to join (and slightly extend) these wider issues at the beginning, as well as introduce afterwards the proposed methodological approach, and leave the local considerations for the end of the introduction (except for a final paragraph stating the aims of the paper, trying again to give a wider view and to describe clear objectives). The text in the current last paragraph related to the economic and environmental significance of Narrangansett Bay could be better suited (and slightly extended) in the discussion. On the other hand, some of the detailed information of the Bay could be moved to the Methods (study site/study method).

As a last minor comment, it’d be appropriate to provide a reference for the statement that *Waterfowl respond to these stressors at the local up to the regional scale and their responses can be apparent nearly immediately and continue after substantial time lags* (4 ^th^ paragraph).

**Methods**

*Study site*

Remove the first sentence from this section (it describes the waterfowl survey, not the study site). Also consider, if necessary, to move some of the information provided in the introduction here, avoiding repetitions.

*Survey method*

Overall, try to be consistent with the verb forms. Most of this section is written in present tense, but some sentences use the past simple tense.

In the 2 ^nd^ paragraph of this section, quote grebes along with ducks, geese and swans (Horned Grebe is also considered). Also in this paragraph, I suggest rewriting the last sentence, to start saying that in most cases birds were identified to species level. For instance: “All birds were identified to the species level, except Lesser and Greater Scaups, that were simple categorized into a single Scaup (Aythya spp.) taxonomic group due to the difficulty of distinguishing them.”

*Data analysis*

Just as a comment, I find that there are too many pre-selected ancillary variables, particularly NAO-related variables. I understand that this has a minor effect, however, as most of them were removed from the final model. On this regard, it’d be worth explaining why a threshold of r = 0.8 was adopted.  

In the third paragraph of this section, I’d suggest to change “for in-depth discussion of methods see (Faith et al., 1987; Minchin, 1987)” to “(for in-depth discussion of methods see Faith et al., 1987; Minchin, 1987)”. Also change “All analysis was conducted in R” to “All analyses were conducted in R”

**Results**

Table 2. Check wording. For instance, at the end of the table caption, change “were not be conducted” to “were not conducted”. I’d suggest changing the last column of the table to %, as this would be more visual for the reader.

The last two sentences of the results’ section seem more appropriate for the discussion. This issue, on the other hand (stability of most sections/changes in section 2 over time) deserve some further attention in the discussion.

**Discussion**

Again I’d slightly change the structure, and try to avoid excessive description. I’d condensate the description of the waterfowl community site by site, trying to avoid references to conservation issues (e.g. implications of highly urbanized areas) here (see below).

I feel a bit uneasy with the sentence “In addition to species’ life history strategies, environmental factors can potentially influence waterfowl distribution in estuaries”. I understand that some environmental factors (such as habitat features) are closely related to life history. So maybe change “environmental” to “climatic”/”meteorological”?

I’d add a paragraph regarding conservation-related issues, including coastal-development issues (i.e. the higher pressure in the inner Bay), as well as other relevant local considerations, plus references to climate change.

Regarding the increase of Hooded Merganser and Bufflehead, why only the latter is discussed? I miss an explanation for the increase of Hooded Merganser too. On the other hand, I miss some more precision regarding the dates of eventsw regarding Bufflehead (e.g. when –roughly- protection measures started to be in place?). And last regarding this issue, I don’t understand why increased protection to buffleheads provides them a competitive advantage in the Bay. They increase in relative numbers, and therefore their rank position changes, but is this an actual advantage in the wintering area?

I appreciate the considerations on the limitations of the study, as described in the penultimate paragraph.

I have read this submission. I believe that I have an appropriate level of expertise to confirm that it is of an acceptable scientific standard, however I have significant reservations, as outlined above.

# Response

**Published:** 2015-09-10

**Authors:** Betty Kreakie

**Affiliations:** US EPA ORD, USA

Thank you so much for taking the time to give us such a thoughtful review. It is clear that your recommendations have greatly improved the text. Below we can included point-by-point responses to your review and have posted a new version based on your comments to F1000Research. Hopefully, this version of the manuscript will be met with your approval. Please let us know if you have any additional concerns or questions. 

 

1. This section is particularly too local it should give a wider view of the work and its potential contribution beyond the study area. The authors propose a methodological approach that could be extended to other areas, to solve similar questions (i.e. how to assess changes in wintering waterfowl communities over time, in a scenario of climate change and increasing coastal development). The last paragraph of the discussion is written under this philosophy, and part of it could be used here. Of course it’s also important to depict the results, but they need to be better contextualized.
**Response:  **We completely agree with this statement.  n fact, early drafts of the manuscript were more focused on developing this method for wide use in environmental monitoring. However, previous reviewers and co-authors felt that we were overreaching and felt it was more interesting to just focus on this particular study area. To compromise, we have added back some text about the broad application of this method.
2. Another issue to address is the excess of repetitions (the same problem is applicable to other sections of the manuscript). For instance, Narragansett Bay is quoted eight times in the abstract alone, and the analytical tool "nonmetric multidimensional scaling" is written in full three times (worth introducing here the acronym NMDS).
**Response: **Thank you for pointing this out. We have rework the abstract (and the remaining text) to remove several of the redundancies and introduced the NMDS acronym to the abstract.
3. Again I find the text too local. Only the first two sentences of paragraph 1, plus paragraph 4 attempt to put the study in a wider context. I suggest to join (and slightly extend) these wider issues at the beginning, as well as introduce afterwards the proposed methodological approach, and leave the local considerations for the end of the introduction (except for a final paragraph stating the aims of the paper, trying again to give a wider view and to describe clear objectives). The text in the current last paragraph related to the economic and environmental significance of Narragansett Bay could be better suited (and slightly extended) in the discussion. On the other hand, some of the detailed information of the Bay could be moved to the Methods (study site/study method).
**Response: **We agree that the previous version of manuscript appears to be very local. As noted in the responses to comment #1, we have added and edited to manuscript to broaden the scope and present the application of the methods. Hopefully the reviewer will feel that we accomplished the goal of broaden the manuscripts scope. Yet coauthors and previous reviewer still feel that this research is in essence simple an analysis of bird communities in Narragansett Bay. We feel that their concern of overreaching is valid. Hopefully with these edits, we have found a balance.
4. As a last minor comment, it’d be appropriate to provide a reference for the statement that *Waterfowl respond to these stressors at the local up to the regional scale and their responses can be apparent nearly immediately and continue after substantial time lags* (4 ^th^ paragraph).
**Response: **This error has been corrected.
5. Remove the first sentence from this section (it describes the waterfowl survey, not the study site). Also consider, if necessary, to move some of the information provided in the introduction here, avoiding repetitions.
**Response: **This edited has been included into the recent version. Due to convention, we have decided to leave some of material about the study area in this section.
6. Overall, try to be consistent with the verb forms. Most of this section is written in present tense, but some sentences use the past simple tense.
**Response: **We have reread this section and made edits as needed.  However, this section does require the both the past and present tense.  Since this survey is ongoing and yet the survey design was completed at the beginning of the study, we used verb tense to indicate which decision were made historically and which still are being used.
7. In the 2 ^nd^ paragraph of this section, quote grebes along with ducks, geese and swans (Horned Grebe is also considered). Also in this paragraph, I suggest rewriting the last sentence, to start saying that in most cases birds were identified to species level. For instance: “All birds were identified to the species level, except Lesser and Greater Scaups, that were simple categorized into a single Scaup (Aythya spp.) taxonomic group due to the difficulty of distinguishing them.”
**Response: **Both of these suggestions have been incorporated. 
8. Just as a comment, I find that there are too many pre-selected ancillary variables, particularly NAO-related variables. I understand that this has a minor effect, however, as most of them were removed from the final model. On this regard, it’d be worth explaining why a threshold of r = 0.8 was adopted.
**Response: **We agree that there were a lot of pre-selected ancillary variables.  We knew that we wanted examine the correlations between climatic and other variables and our NMDS structure, but there was not enough literature on the subject to reduce those variables *a priori*. Since these variables were computational easy to derived, we decided to error on the side of caution. We also change the wording to thresholding to reflect more accurately how we selected variables.
9. In the third paragraph of this section, I’d suggest to change “for in-depth discussion of methods see (Faith et al., 1987; Minchin, 1987)” to “(for in-depth discussion of methods see Faith et al., 1987; Minchin, 1987)”. Also change “All analysis was conducted in R” to “All analyses were conducted in R”
**Response: **We have corrected both of these errors.
10. Table 2. Check wording. For instance, at the end of the table caption, change “were not be conducted” to “were not conducted”. I’d suggest changing the last column of the table to %, as this would be more visual for the reader.
**Response: **This error has been corrected and proportions have been converted to percentages.
11. The last two sentences of the results’ section seem more appropriate for the discussion. This issue, on the other hand (stability of most sections/changes in section 2 over time) deserve some further attention in the discussion.
**Response: **This comment had been completely incorporated into the text. We have deleted the last two sentences from results. This text has been added the discussion along with additional explanation.
12. Again I’d slightly change the structure, and try to avoid excessive description. I’d condensate the description of the waterfowl community site by site, trying to avoid references to conservation issues (e.g. implications of highly urbanized areas) here (see below).
**Response: **This concern has been addressed through the edits to other comments. 
13. I feel a bit uneasy with the sentence “In addition to species’ life history strategies, environmental factors can potentially influence waterfowl distribution in estuaries”. I understand that some environmental factors (such as habitat features) are closely related to life history. So maybe change “environmental” to “climatic”/”meteorological”?
**Response: **We have incorporated this suggestion into the text.
14. I’d add a paragraph regarding conservation-related issues, including coastal-development issues (i.e. the higher pressure in the inner Bay), as well as other relevant local considerations, plus references to climate change.
**Response: ** This concern has been addressed through the additional paragraph in the discussion.
15. Regarding the increase of Hooded Merganser and Bufflehead, why only the latter is discussed? I miss an explanation for the increase of Hooded Merganser too. On the other hand, I miss some more precision regarding the dates of eventsw regarding Bufflehead (e.g. when –roughly- protection measures started to be in place?). And last regarding this issue, I don’t understand why increased protection to buffleheads provides them a competitive advantage in the Bay. They increase in relative numbers, and therefore their rank position changes, but is this an actual advantage in the wintering area?
**Response:  **This paragraph is only intended to speculative about possible reason behind these shifts. We did not explicitly test. It was an interest discovery and we did not want to simply ignore it. We have add some text to this paragraph to it clear that we simply presenting some thoughts about what might be behind this pattern. Clearly more research is needed on this.

# Response

**Published:** 2015-10-22

**Authors:** Betty Kreakie

**Affiliations:** US EPA ORD, USA

I now realize that I should have waited to post the response after the new version was published.  Version 2, which addresses your concerns, is now published.  Sorry for any confusion that this may have caused.

# Peer review: Referee response for version 1

**DOI:** 10.5256/f1000research.6509.r8808

**Published:** 2015-07-09

**Authors:** Nigel Yoccoz

**Affiliations:** 1 Department of Arctic and Marine Biology, University of Tromsø, Tromsø, Norway

**General comments**

Environmental monitoring means different things to different people. It always involves a long-term commitment – that is, a given system is followed up over time, years to decades – but how much this is driven by hypotheses about what drive the changes of the system, and/or about the consequences of these changes, vary. One could use one of the most famous examples of environmental monitoring – the monitoring of CO2 concentrations initiated by Charles David Keeling in 1958 at Hawaii’s Mauna Loa observatory. Keeling was expecting a rise as a consequence of the combustion of fossil fuels, and there were different hypotheses regarding the rate of increase depending on in particular exchange between atmosphere and ocean (see e.g., Revelle and Suess 1957). Keeling concluded for example in 1960 “At the South Pole the observed rate of increase is nearly that to be expected from the combustion of fossil fuel (1.4 p.p.m.), if no removal from the atmosphere takes place” (p. 203; Keeling used measurements from both hemispheres, not just Mauna Loa). This was therefore a strongly hypothesis-driven monitoring – but that did not exclude surprises, in particular the large seasonal variations due to photosynthetic activity. But such surprises often came because there are rather precise expectations about possible changes (see Lindenmayer *et al. *2010 for a related discussion). Many environmental monitoring programs, however, do not have such underlying hypotheses, and are often characterized as being more of the surveillance type (see Nichols and Williams 2006). The main goal is to identify changes – other than due to the stochastic variability of the system. This paper falls in this category – the main purpose is to describe waterfowl communities in a given area, the Narragansett Bay, and identify possible changes (as e.g., an upward trend in two species, Bufflehead and Hooded Merganser). Variables that could drive these changes, and with a relevance for local or regional management, are not included in the analyses – environmental data used refer either to site variables which are unlikely to change (latitude/longitude, bathymetry, area) or that rather reflect short-term temporal variation (wind speed on the day of the survey or just prior to it, North Atlantic Oscillation – a measure of regional atmospheric patterns – in the month of the survey or the months prior to it). That in fact identifying the causes of observed changes in waterfowl communities will be hard without having more explicit hypotheses is made clear in some parts of the discussion. How can we know for example if an observed increase in a wintering duck population is due to 1) improved reproductive success on the breeding grounds, 2) lower hunting pressure in the fall, and 3) change in wintering areas (these hypotheses are not exclusive, so the problem is to assess their relative importance, as for CO2 concentrations: burning fuels, exchange atmosphere-ocean and photosynthetic activity all play a role). Monitoring environmental systems should, in my opinion, aim at characterizing the state of the system but also the drivers of changes (Yoccoz et al. 2001; Yoccoz 2012; see Lindenmayer et al 2015 for a thorough discussion). Doing this without formulating hypotheses (and if possible quantitative models) is difficult. Even if the paper focuses on developing a baseline for environmental monitoring, it also proposes “a statistical framework for future environmental monitoring”. I do not see how the proposed framework will allow for such a quantitative evaluation of hypotheses.

Another concern is the reference to a baseline – using the 2004-2011 data. It is most likely that waterfowl communities during that period have already been heavily impacted by important environmental drivers, either positively (think about the increase of some goose populations) or negatively. Taking those years as a baseline might lead to what Pauly (1995) in the context of fisheries call the “syndrome of shifting baselines” (see Clavero 2014 for an example with non-native species). What we see as a baseline is already very different from historical conditions – irrespective of the reference time used to define “historical”. Monitoring requires we determine the state of the system, but calling it a baseline may be rather misleading without at least a comment.

A final general comment relates to waterfowl as “logical candidates for monitoring environmental health” (p. 2) because they respond to “a wide array of environmental stressors [...] at the local up to the regional scale”. Clearly, what we monitor should reflect the changes we expect, but they should also allow for disentangling causes of change – for example if managers want to act on these causes. Knowing if for example causes are local or regional will determine where policy decisions should be made. Monitoring system variables, such as waterfowl communities, that are influenced by a large number of drivers at very different scales may make them too “integrative” for management (or scientific) purposes. Of course, it may be that there will be a main driver of change (think of DDT and birds of prey) but that seems to be unlikely in this case. I personally think we should aim at monitoring directly state variables and rely less on indicators, but I understand also the practical (and financial) difficulties. But if one relies upon indicators, or surrogates, at least simple conceptual models linking indicators and state variables would help. It would also make transparent assumptions regarding the stability of the links.

To conclude, the authors have done a great job organizing and synthesizing a waterfowl survey over nearly 10 years. The nest step – “future environmental monitoring” – should go further and provides us with a conceptual model of expected changes in the waterfowl community as stressors change at different spatial scales, and if one has the necessary information to assess the relative impact of these stressors.

**Specific comments:**

NMDS is one among many multivariate methods that can be used for analyzing spatio-temporal community data (see Legendre and Gauthier 2014 for a recent review). These methods do not make assumptions about the distribution of the data (p.2), but they all make assumptions about what is a “good” measure of e.g. community similarity (for example one could consider the number of species the two communities share, and how many species that are unique to each community, and combine these numbers in different similarity indices). If one wants to make inference (for the number of axes to be interpreted, or relationships with environmental variables), most analyses rely upon permutation tests which do not rely upon distribution of the data but may be sensitive to dependence among samples. Particularly to identify relationships with environmental variables, alternative approaches to NMDS such as Canonical Correspondence Analysis, would have been interesting to use (ter Braak 1987, Legendre et al. 2011).

p. 4, it is written that the choice of ancillary (environmental) variables was “hypothesis driven”. It would have helped to be more explicit about which component of the variation could be explained by these variables, and in what way it was related to the stressors described e.g. p.2. Clearly the first 10 variables (latitude to wetland area) are unlikely to change during the 8 years of the survey, so they would explain the spatial variation only. Wind speed the day of the survey might explain the day to day variation (but given there is no replicate survey within a year, it will be confounded with the yearly variation), whereas NAO.fall might reflect weather conditions during migration and early winter.

Table 2, p. 5: for counts with skewed distributions, the standard deviation (SD) might be a poor descriptor of the variability (the mean might also be a poor descriptor of location). It might be useful to give the range and median in addition to mean and SD.

Table 3: what is meant by “results”? Correlations? How are P-values calculated?

p. 7: *Bucephal* *a*

p. 8: species’ life history strategies: use traits rather, you do not really investigate strategies here.

I have read this submission. I believe that I have an appropriate level of expertise to confirm that it is of an acceptable scientific standard, however I have significant reservations, as outlined above.

## References

[1] ClaveroM: Shifting Baselines and the Conservation of Non-Native Species. *Conservation Biology*.2014;28(5) : 10.1111/cobi.12266 1434-1436 10.1111/cobi.12266

[2] KeelingCD: The Concentration and Isotopic Abundances of Carbon Dioxide in the Atmosphere. *Tellus*.1960;12(2) : 10.1/j.2153-3490.1960.tb01300.x 200-203 10.1/j.2153-3490.1960.tb01300.x

[3] LegendrePGauthierO: Statistical methods for temporal and space-time analysis of community composition data. *Proc Biol Sci.*.2014;281(1778) : 10.1098/rspb.2013.2728 20132728 10.1098/rspb.2013.2728 24430848PMC3906937

[4] LegendrePOksanenJter BraakCJF: Testing the significance of canonical axes in redundancy analysis. *Methods in Ecology and Evolution*.2010;2(3) : 10.1111/j.2041-210X.2010.00078.x 269-277 10.1111/j.2041-210X.2010.00078.x

[5] LindenmayerDBLikenGEKrebsCJHobbsRJ: Improved probability of detection of ecological “surprises”. *Proc Natl Acad Sci U S A*.2010;107(51) : 10.1073/pnas.1015696107 21957-21962 10.1073/pnas.1015696107 21098660PMC3009814

[6] LindenmayerDBBurnsELTennantPDickmanCRGreenPTKeithDAMetcalfeDJRussell-SmithJWardleGMWilliamsDBossardKdeLaceyCHaniganIBullCMGillespieGHobbsRJKrebsCJLikensGEPorterJVardonM: Contemplating the future: Acting now on long-term monitoring to answer 2050's questions. *Austral Ecology*.2015;40(3) : 10.1111/aec.12207 213-244 10.1111/aec.12207

[7] NicholsJDWilliamsBK: Monitoring for conservation. *Trends Ecol Evol.*.2006;21(12) : 10.1016/j.tree.2006.08.007 668-673 10.1016/j.tree.2006.08.007 16919361

[8] PaulyD: Anecdotes and the shifting base-line syndrome of fisheries. *Trends Ecol Evol.*.1995;10(10) : 10.1016/S0169-5347(00)89171-5 430 10.1016/S0169-5347(00)89171-5 21237093

[9] RevelleRSuessHE: Carbon Dioxide Exchange Between Atmosphere and Ocean and the Question of an Increase of Atmospheric CO2 during the Past Decades. *Tellus*.1957;9(1) : 10.1111/j.2153-3490.1957.tb01849.x 18-27 10.1111/j.2153-3490.1957.tb01849.x

[10] ter BraakCJF: Canonical correspondence analysis: A New Eigenvector Technique for Multivariate Direct Gradient Analysis. *Ecology*.1986;67(5) : 10.2307/1938672 1167-1179 10.2307/1938672

[11] YoccozNG: Ecological Monitoring. *eLS*.2012; 10.1002/9780470015902.a0023571 10.1002/9780470015902.a0023571

[12] YoccozNGNicholsJDBoulinierT: Monitoring of biological diversity in space and time. *Trends Ecol Evol.*.2001;16(8) : 10.1016/S0169-5347(01)02205-4 446-453 10.1016/S0169-5347(01)02205-4

# Peer review: Referee response for version 1

**DOI:** 10.5256/f1000research.6509.r7881

**Published:** 2015-03-30

**Authors:** L. Ignacio Vilchis

**Affiliations:** 1 Wildlife Health Center, School of Veterinary Medicine, University of California Davis, Davis, CA, USA

**General Comments**

This study provides an excellent contribution to our knowledge of baseline species diversity and abundance for the waterfowl community in Narragansett Bay, Rhode Island. The authors analyze longitudinal survey data from the Narragansett Bay Winter Waterfowl Survey (NBWWS) in order to detect shifts in the species composition of the water waterfowl community and to explore if environmental drivers might be driving any change. On a regional scale the study covers almost the entire bay, and considering the general paucity of such monitoring programs, this study is important in that it documents baseline knowledge of the species diversity and abundance of the areas waterfowl and seabird species.

**Specific Comments**

Introduction:

The main purpose of the introduction was to argue the need for baseline monitoring data for waterfowl communities. The authors do achieve this objective in first and fourth paragraphs, leading to the purpose of study in the last sentence of the fifth paragraph of the introduction. However, I did not find all the information in the introduction leading directly to the statement of purpose of the study, with this statement no matching what was stated in the abstract. The second and third paragraphs for example, include information that would be better suited for the methods. Also, while the authors do a great job in explaining why waterfowl are logical candidates for monitoring environmental health, I found the excessive references of the citations in the fourth paragraph a bit distracting. I am not certain of the editorial guidelines of *F1000Research *(I looked these up but failed to find the citation/reference guidelines), but I think a generally accepted rule of thumb is to not list more than three references per citation (although I could very well be wrong here).  Nevertheless, when excessive references and citations are used it distracts the reader from the written message and disrupts the flow of the paragraph. I understand that you have to cite certain statements, but I would advice the authors to go through their citations and only cite essential sources. Doing so along with moving the information from the second and third paragraphs to the methods would help streamline the introduction.

Methods:

A general comment on the survey technique: As written, the survey methodology explains that complete counts are made in each of the areas corresponding to the site locations. It is not clear however, how observers would delineate the off shore boundaries of the survey areas, particularly those facing each other or off shore boundaries in general. How were these distances discerned? I am curious to how the observers gauged the distance from shore so that birds in the survey area from the opposite shoreline were not recounted. Did they use range finders? Adding some text explaining this would benefit the methods.

I downloaded the data that is made available with this study via the *F1000Research *website and found that in the data set describing the winter waterfowl survey ( 10.5256/f1000research.6080.d42954) the column corresponding to buffleheads is missing. Was a different dataset used for this study? If not, why are buffleheads not included in the dataset but in the result of the study?

First paragraph: In the fourth sentence the authors refer to “This area”. I would suggest clearing up what 'area' is being referred to. Also, in the fifth sentence the authors state that the southern portion of the bay is deeper. However, they did not mention deeper ‘than what’. Typically the reader expects to see the full comparison. I know these are nit picky grammar and style comments, but clearing these two minor points would make the paragraph read much better.

Fourth paragraph: In the first sentence the authors start by saying “Counting is completed” when the rest of the verbs in the paper are in the past tense. I think changing this to ‘counting was completed’ would be better. In the second sentence, the authors explain: “Every bird seen on the water surface or on the adjoining shoreline up to 50 m from the water is counted; when possible sex and age were also noted”. Is this vertically or horizontally from the water? I am assuming that they mean 50 meters from the waters edge or shoreline – or along the coast.  Please state this more clearly. 

Seventh paragraph: Please explain why the data was transformed and why the Wisconsin double transformation was chosen. Because non-metric multidimensional scaling uses rank orders, it is not clear why the transformation was used. Stating this more clearly in the text would benefit the paper.

Eighth paragraph. In the last sentence, I would suggest using ‘>’ instead of ‘greater than’.

Results:

It is not obvious to the reader why the data presented in figures 1 & 2 show survey data up to 2012, while the ordination only used data up to 2011. Stating this reason more clearly in the methods would benefit the paper.

The legend of Table 2 is not clear. Changing the wording could help clear up the message of the legend. For example, the last two sentences state: “*Species making up less than 1% of the community, and considered rare. Trend analysis (Figure 3) were not be conducted on these rare species.” The tense is wrong here, please change analysis to analyses. Also I would suggest saying this a bit clearer using fewer words, with perhaps something like this? “Species with overall species compositions < 1% were considered rare and excluded from trend analysis”. On this note, if I understand correctly, the legend states that trend analyses were not conducted on rare species and then refers to Figure 3. Figure 3 however shows trends in rank abundance of all 23 species listed in Table 2. This is confusing. Clearing this up in the legend or text would benefit the paper.

Regarding the non-metric multidimensional scaling (NMDS), why were four dimensions chosen over two or three? Did solutions with fewer dimensions have too large stress value? Was this assessed with a scree plot?  Stating these details would benefit the paper and help the reader understand why four dimensions were the best choice for the NMDS analysis. Furthermore, in Figure 4, where the results of the NMDS are first shown, it is not clear why are king eiders and long-tailed ducks not shown in the plot. I am assuming from table two that this is because these two species have proportions of the total waterfowl community abundance that are close to zero. Using this logic however, barrow’s goldeneyes should also be excluded from the plot—but they are not. As the NMDS analysis is the central component of the study, the authors should either include all 23 species or clearly state why only 20 were used. As is, the results are somewhat confusing and distract the reader from the overall message of the study.

The authors use Figure 5 to detect shifts in the waterfowl community structure of Narragansett Bay during the 8-yr period of the study (2004 and 2011). It is confusing why the NMDS plots in Figure 5 are different to those in Figure 4. I might be missing something, but should not the ordination plots in Figure 5 have the same points and axes as in Figure 4? Are these plots showing the other two dimensions of the ordination?  The details of Figure 5 should be made more elaborate explanation in the text. I am assuming the plots in Figure 5 show the average ordination positions for each section-year combination, with these being color coded for each year. Instead of this exercise, I would suggest authors use analysis of similarity (ANOSIM) to tests hypotheses of community change in each of the sections or the entire bay throughout the 8-yr study period.  In this way the authors could obtain an objective measure indicating if there has been community change (or no change) in the waterfowl of Narragansett Bay during the 8-yr study period. Using ANOSIM here would be analogous to using ANOVAs to assess before and after changes at certain breaks in the 8-yr study period.  ANOSIM however, uses the same similarity matrices imputed into NMDS and is philosophically allied with NMDS ordination. The functions need to perform this analysis are included in the R vegan package, which the authors have shown to have an excellent handle on using. Including ANOSIM in the study to objectively assess temporal changes in the beta diversity of the Narragansett Bay waterfowl community would therefore fit in nicely in the study as as it complements NMDS.

In the last paragraph of the results the authors state: “it is thought that waterfowl communities in this part of the Bay vary greatly according to the intensity of winter and amount of ice”. This statement should have a citation referring to the reference thinking or hypothesizing this mechanism. 

Discussion:

First paragraph. In the first sentence, I would suggest replacing “expected” for “baseline”. In the third sentence the authors refer to NMDS as a monitoring tool. NMDS is an ordination analysis used to interpret ecological data, and not a monitoring tool. I think this statement could be reworded better saying for example: “Using NMDS to analyzing monitoring data is a valuable approach to assess…”

Sixth paragraph: Again, NMDS is not an approach—it is an analytical tool. Using this ordination technique is the approach the authors took to assess the community structure of the Narragansett Bay waterfowl community. Addressing and fixing this accordingly, will benefit the flow of the text.

The second objective of the of the study was to explore if environmental drivers that might be driving changes in the species composition of the waterfowl species composition. I think this objective needs to be addressed a bit more in the discussion. It is only touched upon lightly. Furthermore, what do the authors think is the reason why they found strong correlations of the average wind speed for the three days prior to survey, area, mean bathymetry of site and latitude with the ordination, and yet the wintering waterfowl species composition of the bay seemed to remain stable through time.

I have read this submission. I believe that I have an appropriate level of expertise to confirm that it is of an acceptable scientific standard, however I have significant reservations, as outlined above.

# Response

**Published:** 2015-10-23

**Authors:** Betty Kreakie

**Affiliations:** US EPA ORD, USA

We greatly appreciate your feedback on our manuscript.  Your thorough and thoughtful review has allowed us to significantly improve the manuscript.  Below are point-by-point responses to your comments.  Your concerns will be reflected in version 3 of the manuscript.  Thank you again for all your help.  Please let us know if you have any additional concerns. 

**1. The main purpose of the introduction was to argue the need for baseline monitoring data for waterfowl communities. The authors do achieve this objective in first and fourth paragraphs, leading to the purpose of study in the last sentence of the fifth paragraph of the introduction. However, I did not find all the information in the introduction leading directly to the statement of purpose of the study, with this statement no matching what was stated in the abstract. The second and third paragraphs for example, include information that would be better suited for the methods.**

Response: This concern was also expressed by the first reviewer of the manuscript.  For version 2, we added considerable text to the introduction about how this method could have broader application.  In the first version, you are correct in suggesting this text should be moved to the Methods section.  However, the second version expands the statement of purpose.  Hopefully these edits will address your concern. 

**2. Also, while the authors do a great job in explaining why waterfowl are logical candidates for monitoring environmental health, I found the excessive references of the citations in the fourth paragraph a bit distracting. I am not certain of the editorial guidelines of *F1000Research *(I looked these up but failed to find the citation/reference guidelines), but I think a generally accepted rule of thumb is to not list more than three references per citation (although I could very well be wrong here).  Nevertheless, when excessive references and citations are used it distracts the reader from the written message and disrupts the flow of the paragraph. I understand that you have to cite certain statements, but I would advice the authors to go through their citations and only cite essential sources. Doing so along with moving the information from the second and third paragraphs to the methods would help streamline the introduction.**

Response: We completely agree that the amount of references were distracting. We have selectively reduced the number of citations in this part.  Specifically, we removed several of the older citations. Most of the material from the older works was more comprehensively covered in the more recent work. 

**3. As written, the survey methodology explains that complete counts are made in each of the areas corresponding to the site locations. It is not clear however, how observers would delineate the off shore boundaries of the survey areas, particularly those facing each other or off shore boundaries in general. How were these distances discerned? I am curious to how the observers gauged the distance from shore so that birds in the survey area from the opposite shoreline were not recounted. Did they use range finders? Adding some text explaining this would benefit the methods.**

Response: To clarify this issue, we added text the first paragraph of the Survey Method subsection of the Methods.

**4. I downloaded the data that is made available with this study via the *F1000Research *website and found that in the data set describing the winter waterfowl survey (** **10.5256/f1000research.6080.d42954** **) the column corresponding to buffleheads is missing. Was a different dataset used for this study? If not, why are buffleheads not included in the dataset but in the result of the study?**

Response:  The wrong data set did get uploaded to the F1000Research page.  Thank you for pointing out this mistake. The correct data are now available.

**5. First paragraph: In the fourth sentence the authors refer to “This area”. I would suggest clearing up what 'area' is being referred to.**

Response: We have changed to this test to “The land surrounding the northern portion of Narragansett Bay.”

**6. Also, in the fifth sentence the authors state that the southern portion of the bay is**

**deeper. However, they did not mention deeper ‘than what’. Typically the reader expects to see the full comparison. I know these are nit picky grammar and style comments, but clearing these two minor points would make the paragraph read much better.**

Response: We have added ”than the portion near Providence, Rhode Island.”  Also, we completely agree that this paragraph now reads much better.

**7. In the first sentence the authors start by saying “Counting is completed” when the rest of the verbs in the paper are in the past tense. I think changing this to ‘counting was completed’ would be better.**

Response: This error has been corrected.

**8. In the second sentence, the authors explain: “Every bird seen on the water surface or on the adjoining shoreline up to 50 m from the water is counted; when possible sex and age were also noted”. Is this vertically or horizontally from the water? I am assuming that they mean 50 meters from the waters edge or shoreline – or along the coast.  Please state this more clearly.**

Response: We have incorporated this recommendation into the text.

**9. Please explain why the data was transformed and why the Wisconsin double transformation was chosen. Because non-metric multidimensional scaling uses rank orders, it is not clear why the transformation was used. Stating this more clearly in the text would benefit the paper.**

Response: We have added text to clarify why this transformation was performed.

**10.  In the last sentence, I would suggest using ‘>’ instead of ‘greater than’.**

Response: This change was added to the text.

**11. It is not obvious to the reader why the data presented in figures 1 & 2 show survey data up to 2012, while the ordination only used data up to 2011. Stating this reason more clearly in the methods would benefit the paper.**

Response:  At the time of the analysis, all the environmental variables were not available through 2012.  The waterfowl survey for 2012 was available and had gone through our QA/QC process.  We decided to include another year into the population, since we were specifically looking for significant trends.  We have added clarification to the text about this issue.

12. The legend of Table 2 is not clear. Changing the wording could help clear up the message of the legend. For example, the last two sentences state: “*Species making up less than 1% of the community, and considered rare. Trend analysis (Figure 3) were not be conducted on these rare species.” The tense is wrong here, please change analysis to analyses. Also I would suggest saying this a bit clearer using fewer words, with perhaps something like this? “Species with overall species compositions < 1% were considered rare and excluded from trend analysis”. On this note, if I understand correctly, the legend states that trend analyses were not conducted on rare species and then refers to Figure 3. Figure 3 however shows trends in rank abundance of all 23 species listed in Table 2. This is confusing. Clearing this up in the legend or text would benefit the paper.

Response:  We apologize for the confusion.  These was a change in approach that obviously did not get corrected in all parts of the final manuscript.  Previous reviewers request that all species be presented in Figure 3 and therefor that figure was edited accordingly.  The manuscript has been corrected to reflect the presented data.

**13. Regarding the non-metric multidimensional scaling (NMDS), why were four dimensions chosen over two or three? Did solutions with fewer dimensions have too large stress value? Was this assessed with a scree plot?  Stating these details would benefit the paper and help the reader understand why four dimensions were the best choice for the NMDS analysis.**

Response:  We explained our process for selecting the number of dimensions in the Methods (third paragraph of the Data Analysis subsection).  In addition, we added text to the Results (second paragraph). 

**14. Furthermore, in Figure 4, where the results of the NMDS are first shown, it is not clear why are king eiders and long-tailed ducks not shown in the plot. I am assuming from table two that this is because these two species have proportions of the total waterfowl community abundance that are close to zero. Using this logic however, barrow’s goldeneyes should also be excluded from the plot—but they are not. As the NMDS analysis is the central component of the study, the authors should either include all 23 species or clearly state why only 20 were used. As is, the results are somewhat confusing and distract the reader from the overall message of the study.**

Response: All 23 species were indeed used to construct the plot.  Both long-tailed duck and king eider plot very low on the y-axis.  Since this is a very complicated plot, we decided to zoom in slightly to have a better view of the main cluster.  This also allowed us to make our labels more legible.  However, we failed to explain this in text.  We have added text to the figure capture to explain this decision and to report values for long-tailed duck and king eider.

**15. The authors use Figure 5 to detect shifts in the waterfowl community structure of Narragansett Bay during the 8-yr period of the study (2004 and 2011). It is confusing why the NMDS plots in Figure 5 are different to those in Figure 4. I might be missing something, but should not the ordination plots in Figure 5 have the same points and axes as in Figure 4? Are these plots showing the other two dimensions of the ordination?  The details of Figure 5 should be made more elaborate explanation in the text.**

Response:  The subplots in figure 5 are the same plots as in figure 4.  For figure 5 we greyed out the site locations, and added the average section location with a square symbol.  However, as you pointed out, this is not intuitively clear.  We added text in the figure caption to clarify this issue.

**16. I am assuming the plots in Figure 5 show the average ordination positions for each section-year combination, with these being color coded for each year. Instead of this exercise, I would suggest authors use analysis of similarity (ANOSIM) to tests hypotheses of community change in each of the sections or the entire bay throughout the 8-yr study period.  In this way the authors could obtain an objective measure indicating if there has been community change (or no change) in the waterfowl of Narragansett Bay during the 8-yr study period. Using ANOSIM here would be analogous to using ANOVAs to assess before and after changes at certain breaks in the 8-yr study period.  ANOSIM however, uses the same similarity matrices imputed into NMDS and is philosophically allied with NMDS ordination. The functions need to perform this analysis are included in the R vegan package, which the authors have shown to have an excellent handle on using. Including ANOSIM in the study to objectively assess temporal changes in the beta diversity of the Narragansett Bay waterfowl community would therefore fit in nicely in the study as it complements NMDS.**

Response: We agree that ANOSIM would tell us if the groups are different and different by year.  In figure 5 we are trying to show how the sections change through the years in context to the of the entire Bay waterfowl community.  Hopefully, the additional text will clarify our intent to the audience.  Additional, we added considerable text to the discussion in version 2 that adds to the interpretation of this figure. 

**17. In the last paragraph of the results the authors state: “it is thought that waterfowl communities in this part of the Bay vary greatly according to the intensity of winter and amount of ice”. This statement should have a citation referring to the reference thinking or hypothesizing this mechanism.** 

Response: This material was moved to the Discussion in version 2.  We have added a citation

to this state.

**18.  In the first sentence, I would suggest replacing “expected” for “baseline”.**

Response: This concern was corrected in version 2.

**19.  In the third sentence the authors refer to NMDS as a monitoring tool. NMDS is an ordination analysis used to interpret ecological data, and not a monitoring tool. I think this statement could be reworded better saying for example: “Using NMDS to analyzing monitoring data is a valuable approach to assess…”**

Response: In version 2, we added text to clarify how we feel that this method could have broader appeal.  However, we do agree that the NMDS is an ordination analysis.  We have edited the text to address this concern.

**20.  Again, NMDS is not an approach—it is an analytical tool. Using this ordination technique is the approach the authors took to assess the community structure of the Narragansett Bay waterfowl community. Addressing and fixing this accordingly, will benefit the flow of the text.**

Response: See above for explanation about how this issue was corrected.

**21. The second objective of the study was to explore if environmental drivers that might be driving changes in the species composition of the waterfowl species composition. I think this objective needs to be addressed a bit more in the discussion. It is only touched upon lightly.**

Response:  The first reviewer of the manuscript agreed with your concern.  Therefore we add material to the Discussion in the second version to further explore our interpretation on the environmental drivers.

**22. Furthermore, what do the authors think is the reason why they found strong correlations of the average wind speed for the three days prior to survey, area, mean bathymetry of site and latitude with the ordination, and yet the wintering waterfowl**

**species composition of the bay seemed to remain stable through time**.

Response: As mentioned above, we did add text to Discussion about these issues.
